# Comparative genomic analysis of the DUF71/COG2102 family predicts roles in diphthamide biosynthesis and B12 salvage

**DOI:** 10.1186/1745-6150-7-32

**Published:** 2012-09-26

**Authors:** Valérie de Crécy-Lagard, Farhad Forouhar, Céline Brochier-Armanet, Liang Tong, John F Hunt

**Affiliations:** 1 Department of Microbiology and Cell Science, University of Florida, Gainesville, FL, 32611, USA; 2 Department of Biological Sciences, Columbia University, Northeast Structural Genomics Consortium, 1212 Amsterdam Ave, New York, NY, 10027, USA; 3 Université de Lyon; Université Lyon 1; CNRS; UMR5558, Laboratoire de Biométrie et Biologie Evolutive, 43 boulevard du 11 Novembre 1918, Lyon, Villeurbanne, F-69622, France

**Keywords:** Diphthamide, Vitamin B12, Amidotransferase, Comparative genomics

## Abstract

**Background:**

The availability of over 3000 published genome sequences has enabled the use of comparative genomic approaches to drive the biological function discovery process. Classically, one used to link gene with function by genetic or biochemical approaches, a lengthy process that often took years. Phylogenetic distribution profiles, physical clustering, gene fusion, co-expression profiles, structural information and other genomic or post-genomic derived associations can be now used to make very strong functional hypotheses. Here, we illustrate this shift with the analysis of the DUF71/COG2102 family, a subgroup of the PP-loop ATPase family.

**Results:**

The DUF71 family contains at least two subfamilies, one of which was predicted to be the missing diphthine-ammonia ligase (EC 6.3.1.14), Dph6. This enzyme catalyzes the last ATP-dependent step in the synthesis of diphthamide, a complex modification of Elongation Factor 2 that can be ADP-ribosylated by bacterial toxins. Dph6 orthologs are found in nearly all sequenced Archaea and Eucarya, as expected from the distribution of the diphthamide modification. The DUF71 family appears to have originated in the Archaea/Eucarya ancestor and to have been subsequently horizontally transferred to Bacteria. Bacterial DUF71 members likely acquired a different function because the diphthamide modification is absent in this Domain of Life. In-depth investigations suggest that some archaeal and bacterial DUF71 proteins participate in B12 salvage.

**Conclusions:**

This detailed analysis of the DUF71 family members provides an example of the power of integrated data-miming for solving important “missing genes” or “missing function” cases and illustrates the danger of functional annotation of protein families by homology alone.

**Reviewers’ names:**

This article was reviewed by Arcady Mushegian, Michael Galperin and L. Aravind.

## Background

**Figure 1:**
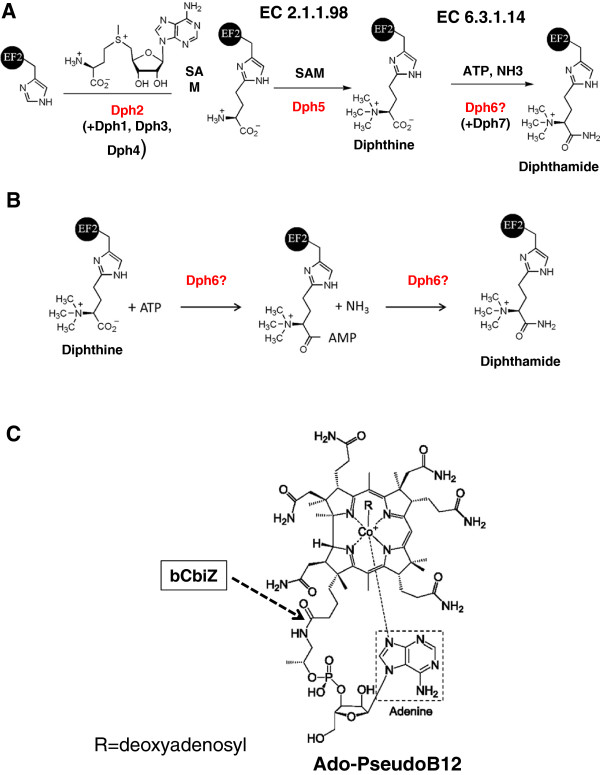
**Structures of diphthamide and B12 precursors and derivatives. **(**A**) The core diphthamide pathway is predicted to contain three enzymes Dph2, Dph5 and Dph6 in Archaea. The formation of diphthine has been reconstituted *in vitro* using Dph2 and Dph5 from *Pyrococcus horikoshii *[4,5]. The enzyme family catalyzing the last step in Archaea and Eukarya Dph6 was missing. In yeast, the first and last steps require additional proteins (Dph1, Dph3 and Dph7). (**B**) Predicted Dph6-catalyzed reactions. (**C**) Ado-Pseudo-B12 structure and hydrolysis site by the bacterial CbiZ enzyme (bCbiZ). Parts (**A**) and (**B**) are adapted with permission from Xuling Zhu; Jungwoo Kim; Xiaoyang Su; Hening Lin; *Biochemistry * 2010, 49, 9649–9657. Copyright 2010 American Chemical Society.

In both Archaea and Eucarya, the translation Elongation Factor 2 (EF-2) harbors a complex post-translational modification of a strictly conserved histidine (His_699 _in yeast) called diphthamide [1]. This modification is the target of the diphtheria toxin and the *Pseudomonas* exotoxin A, which inactivate EF-2 by ADP-ribosylation of the diphthamide [2,3]. Although the diphthamide biosynthesis pathway was described in the early 1980′s [2,3], the corresponding enzymes have only recently been characterized. *In vitro* reconstitution experiments have shown that the first step, the transfer of a 3-amino-3-carboxypropyl (ACP) group from *S*-adenosylmethionine (SAM) to the C-2 position of the imidazole ring of the target histidine residue, is catalyzed in Archaea by the iron-sulfur-cluster enzyme, Dph2 [4,5] (Figure 1A). Genetic and complementation studies have shown that the catalysis of the same first step requires four proteins (Dph1-Dph4) in yeast and other eukaryotes [6-9]. The subsequent step, trimethylation of an amino group to form the diphthine intermediate, is catalyzed by diphthine synthase, Dph5 (EC 2.1.1.98) (Figure 1A) [10,11]. The last step, the ATP-dependent amidation of the carboxylate group [12], is catalyzed by diphthine-ammonia ligase (EC 6.3.1.14), but the corresponding gene has not been identified (http://www.orenza.u-psud.fr/). A protein involved in this last step was recently identified in yeast (YBR246W or Dph7), but it is most certainly not directly involved in catalysis as it is not conserved in Archaea and it contains a WD-domain likely to be involved in protein/protein interactions [13].

Using a combination of comparative genomic approaches, we set out to identify a candidate gene for this orphan enzyme family. Based on taxonomic distribution, domain organization of gene fusions, physical clustering on chromosomes, atomic structural data, co-expression, and phenotype data, a promising candidate was identified, the family called Domain of Unknown Function family DUF71(IPR002761) in Interpro [14]. This family is also called ATP_bind_4 (PF01902) in Pfam [15]or Predicted ATPases of PP-loop superfamily (COG2102) in the Cluster of Ortholous Group database [16]. However, detailed analysis of the DUF71 family revealed that this family is almost surely not isofunctional. Some Archaea contain two very divergent copies of the gene, while homologs are found in Bacteria, which are known to lack diphthamide. This observation suggests that some DUF71 members have different functions and probably participate in different biochemical pathways.

## Methods

### Comparative genomics

The BLAST tools [17] and resources at NCBI (http://www.ncbi.nlm.nih.gov/) were routinely used. Multiple sequence alignments were built using ClustalW [18] or Multialin [19]. Protein domain analysis was performed using the Pfam database tools (http://pfam.janelia.org/) [15]. Analysis of the phylogenetic distribution and physical clustering was performed in the SEED database [20]. Results are available in the “Diphthamide biosynthesis” and “DUF71-B12” subsystem on the public SEED server (http://pubseed.theseed.org/SubsysEditor.cgi). Phylogenetic profile searches were performed on the IMG platform [21] using the phylogenetic query tool (http://img.jgi.doe.gov/cgi-bin/w/main.cgi?section=PhylogenProfiler&page=phyloProfileForm). Physical clustering was analyzed with the SEED subsystem coloring tool or the Seedviewer Compare region tool [20] as well as on the MicrobesOnline (http://www.microbesonline.org/) tree based genome browser [22]. The SPELL microarray analysis resource [23] was used through the *Saccharomyces* Genome Database (SGD) (http://www.yeastgenome.org/)[24] to analyze yeast gene coexpression profiles. Clustering of yeast deletion mutants based on phenotype analysis was analyzed through the yeast fitness database available at http://fitdb.stanford.edu/[25,26]. Mapping of gene distribution profile to taxonomic trees were generated using the iTOL suite (http://itol.embl.de/index.shtml) [27]. Sequence logos were derived using the WebLogo platform [28].

### Structure analysis

Visualization and comparison of protein structures and manual docking of ligand molecules were performed using PyMol (The PyMOL Molecular Graphics System, Version 1.4.1, Schrödinger, LLC). XtalView [7] was used for the protein docking exercises.

### Phylogenetic analyses

The survey of the 1996 complete prokaryotic genomes available at the NCBI (http://www.ncbi.nlm.nih.gov/) using BLASTP [17] (default parameters) allowed identification of 119 bacterial and 144 archaeal DUF71 homologs in addition to the 182 eukaryotes homologs identified in the RefSeq database at the NCBI [29] (Additional file 1: Table S1). The retrieved sequences were aligned using MAFFT [8] and the resulting alignment was visually inspected using ED, the alignment editor of the MUST package [30]. The phylogenetic analysis of the 445 sequence was performed using the neighbor-joining distance method implemented in SeaView [31]. The robustness of the resulting tree was assessed by the non-parametric bootstrap method (100 replicates of the original dataset) implemented in SeaView. A second phylogenetic analysis restricted to 50 archaeal and eukaryotic homologs representative of the genetic and genomic diversity of these two Domains was performed using the Bayesian approach implemented in Phylobayes [6] with a LG model.

## Results and discussion

### Comparative genomics points to DUF71/COG2102 as a strong candidate for the missing diphthamide synthase family

**Figure 2:**
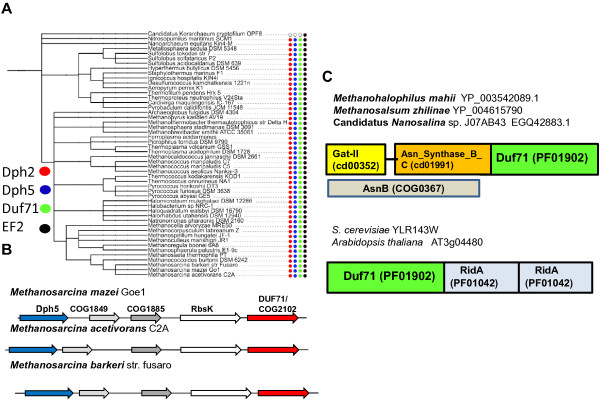
**Comparative genomic analysis of the DUF71 family. **(**A**) Distribution of the core diphthamide genes Dph2 and Dph5 and of EF-2 and DUF71 in Archaea, according to data derived from the “Diphthamide biosynthesis“ subsystem in the SEED database. The tree is a species tree constructed in iTol (itol.embl.de/). The presence and absence of the specific genes was derived from the “Diphthamide biosynthesis“ subsystem. (**B**) Physical clustering of DUF71/COG2102 genes with Dph5 in three *Methanosarcina *genomes derived from the MicrobesOnline database (http://www.microbesonline.org/). (**C**) Examples of proteins containing domains fused to DUF71 in Archaea and Eucarya. Accession numbers and COG, CDD, or Pfam domain numbers are given in parentheses.

The distribution of known diphthamide biosynthesis genes in Archaea was analyzed using the SEED database and its tools [20]. The 59 archaeal genomes analyzed all contained an EF-2 encoding gene. Analysis of the distribution of Dph2 and Dph5 in Archaea showed that 58/59 genomes encoded these two proteins. The only archaeon lacking both Dph2 and Dph5 was *Korarchaeum cryptofilum* OPF8 (Figure 2A). We therefore hypothesized that this organism has lost the diphthamide modification pathway even if the *K. cryptofilum* EF-2 still harbors the conserved His residue at the site of the modification (His_603 _in the *K. cryptofilum* sequence*,* Accession B1L7Q0 in UniprotKB). Using the IMG/JGI phylogenetic query tools [21], we searched for protein families found in all Archaea except *Korarchaeum cryptofilum* OPF8, present in *Saccharomyces cerevisiae* and *Homo sapiens* but absent in *Escherichia coli* and *Bacillus subtilis*, as bacteria are known to lack this modification pathway. Only one family, DUF71/COG2102, followed this taxonomic distribution. This family had been described previously as a PP-loop ATPase of unknown function containing a Rossmannoid class HUP domain [32].

Using the neighborhood analysis tool of the SEED database [20], physical clustering was generally not observed between the *dph2*, *dph5* and *DUF71* genes except in three *Methanosarcina* genomes where the *dph5* is located in the vicinity of *DUF71* genes (Figure 2B). If members of the DUF71 catalyze the last step of diphthamide synthesis they should bind ATP [12]. Structural analysis of the DUF71 protein from *Pyrococcus furiosus* (PF0828) reveals the presence of two distinct domains: an N-terminal HUP domain that contains a highly conserved PP-motif that interacts with ATP (PDB id: 3RK1) and AMP (PDB id: 3RK0), and a C-terminal 100-residue domain belonging to a novel fold with a highly conserved motif GEGGEF/YE_188_T/S (*P*. *furiosus* numbering) that is probably involved in substrate binding and recognition [33].

### Coexpression, phenotype and structural data link the yeast DUF71 to translation and diphthamide biosynthesis

YLR143w is the only *S*. *cerevisiae* DUF71 family member. Using YLR143w as input in the SPELL co-expression query tool [23] showed that nearly all co-expressed genes were involved in translation and ribosome biogenesis (Additional file 2: Table S2). This observation suggested that the DUF71 protein family has a role in translation as expected for a protein modifying EF-2. Like all known diphthamide synthesis genes, *YLR143w* is also not essential. More specifically, deletion of any of the five known diphthamide genes confers sordarin resistance in yeast [34,35] and *ylr143w*Δ strain was shown to be as resistant to this compound as the diphthamide deficient strains (see supplemental data in [34]). Furthermore, in a recent complete analysis of relationships between gene fitness profiles (co-fitness) and drug inhibition profiles (co-inhibition) from several hundred chemogenomic screens in yeast [25,26] available at http://fitdb.stanford.edu/, it was found that among the top ten interactors with YLR143w by homozygous co-sensitivity are DPH5, DPH2, DPH4 (or JJJ3) and the newly identified DPH7 (or YBR246w) (Additional file 3: Figure S1). Both the coexpression and phenotype data thereby strongly support the hypothesis that YLR143w catalyzes the missing last step of diphthamide biosynthesis, even if one cannot rule out at this stage that other catalytic subunits yet to be identified may also be required.

**Figure 3:**
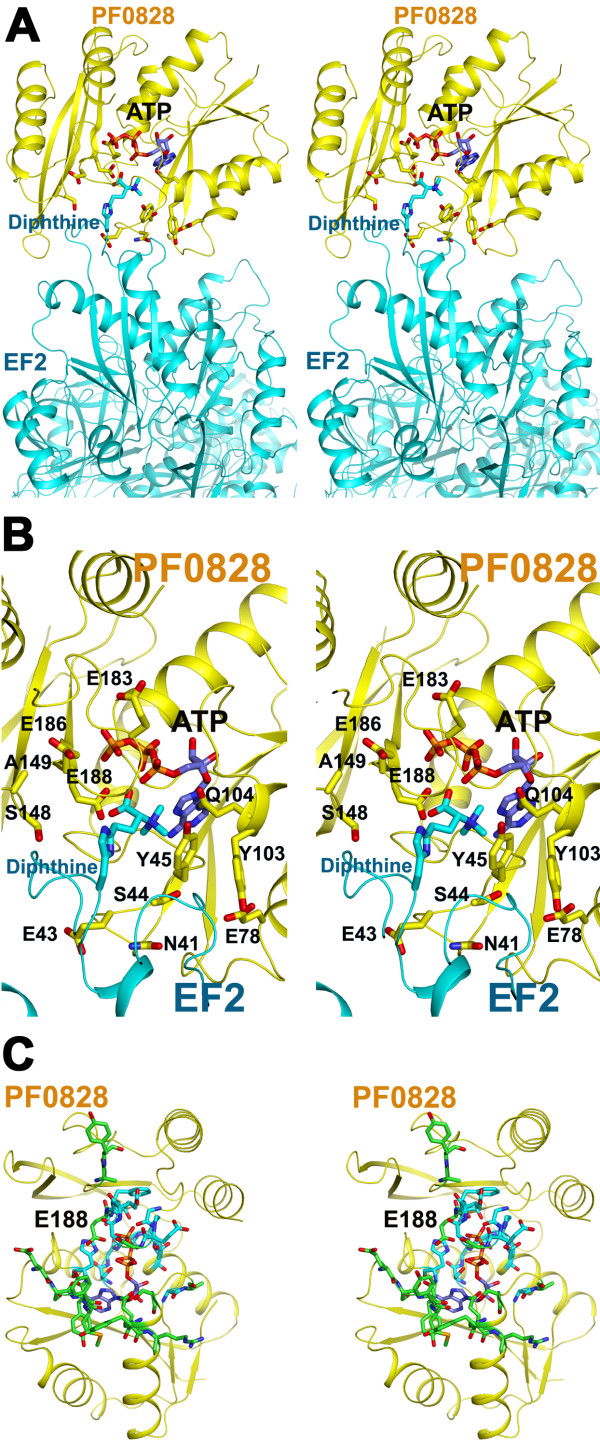
**Structural analysis of the DUF71 (PF0828) putative activesite. **(**A**) Docking of modified EF-2 (cyan, PDB id: 3B82) onto ATP-bound structure of PF0828 (yellow, PDB id: 3RK1). ATP and several residues of PF0828 (DUF71), which are conserved among archaeal and eukaryotic orthologs, and diphthine of EF-2 (see text for details) are shown in stick models. (**B**) Close-up stereo pair of panel A. Diphthine of EF-2 and the side chains of conserved residues of PF0828, at the interface of PF0828 and EF-2, are shown in stick models and labeled. (**C**) Stereo pair view of ATP-binding region of PF0828. Residues that are conserved among Dph6 and DUF71-B12 families are depicted in stick models with carbon atoms in cyan, while the residues that are specific to Dph6 family are shown in stick models with carbon atoms in green. Oxygen and nitrogen atoms are shown in red and blue in all stick models, respectively.

Finally, comparison of ATP- and AMP-containing structures of PF0828 reveals that the active site of the former has a narrow groove at the end of which only the α-phosphate of ATP is exposed to the solvent whereas the active site of the latter is wide open (Figure 3A and B). Also, there is a sharp turn at the α-phosphate of ATP, suggesting that it is the site of the nucleophilic attack. We therefore performed a docking exercise using the EF-2 structure (PDB id: 3B82) [36] with the ATP-containing structure of PF0828. The docking revealed that the active site groove of the ATP-containing structure can easily accommodate diphthine with a few minor clashes between the two structures (Figure 3A and B).

The modeling also showed that the carboxyl group of diphthine resides near the α-phosphate of ATP and carboxylate group of residue Glu_188_, suggesting that nucleophilic attack by diphthine on the α-phosphate of ATP is highly feasible (Figure 3B). As shown in Figure 3B, the modelling also shows that several residues which are highly conserved among archaeal and eukaryotic PF0828 and YLR143w orthologs beside E_188_, including S_44_, Y_45_, E_78_, Y_103_, Q_104_, A_149_, E_183 _and E_186 _(Additional file 3: Figure S2), are at the interface of the modelled complex of PF0828 with EF-2, supporting the hypothesis that they play important roles in EF-2 recognition (Figure 3B).

### Linking DUF71 family members to ammonia transfer reactions

The diphthine ammonia lyase reaction requires a source of NH_3_[12]. Domain fusions involving members of the DUF71 family in the Pfam database [15] suggests the source of NH_3 _might vary depending on the organism. For example, in a few Archaea (e.g. *Methanohalophilus mahii* DSM 5219, *Methanosalsum zhilinae* DSM 4017 or ‘*Candidatus* Nanosalinarum sp. J07AB56′), a COG0367/AsnB asparagine synthetase [glutamine-hydrolyzing] (EC 6.3.5.4) domain is found at the N-terminus of the DUF71 domain (Figure 2C). This AsnB domain can be further separated into two subdomains, an N-terminal class-II glutamine amidotransferase domain (GAT-II) [37] and an Asn_Synthase_B_C PP-loop ATPase domain (Figure 2C) . This domain organization suggests that in this subset of enzymes, the hydrolysis of glutamine catalyzed by the GAT-II domain could provide the NH_3 _moiety to both the DUF71 and the Asn_Synthase_B_C enzymes. On the other hand, in many eukaryotes such as yeast and *Arabidopsis thaliana*, two YjgF-YER057c-UK114-like domains are fused to the C-terminus of the DUF71 protein as previously noted by Aravind et al. [32] (Figure 2C). The stand-alone members of the YjgF-YER057c-UK114 family, now called the RidA family (for reactive intermediate/imine deaminase A), have been shown to deaminate products generated by PLP-dependent enzymes, which results in the release of NH_3_[38]. The RidA domains fused to DUF71 could therefore be involved in providing the NH_3_ ammonium moiety for diphthamide synthesis.

### The Duf71 family is not monofunctional

**Figure 4:**
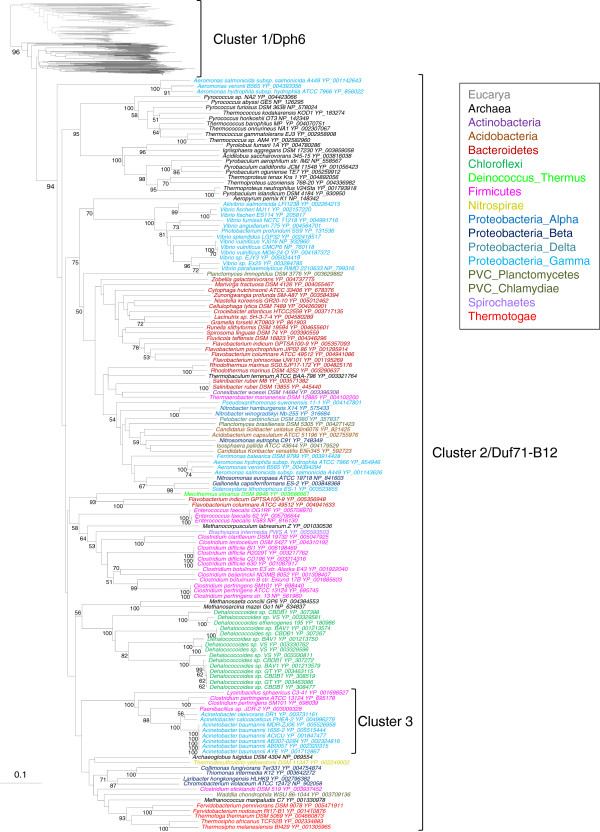
**Neighbor-joining phylogenetic tree of the 445 DUF71 homologs identified in public databases. **The scale bar represents the average number of substitutions per site. Numbers at nodes are bootstrap values. For clarity only values greater than 50% are indicated. Colors correspond to the taxonomic affiliation of sequences (see the box on the figure for details). The full tree of Cluster 1 is shown in Additional file 3: Figure S3).

The taxonomic distribution of DUF71 homologs in available complete genomes confirmed that DUF71 is present in one or occasionally two copies in all Archaea except the korarchaeon *K*. *cryptofilum* (Table 1 and Additional file 1: Table S1). This pattern is consistent with an ancient origin of the DUF71 gene in Archaea. In sharp contrast, DUF71 is sporadically distributed in Bacteria, being present only in a few representatives of some phyla (Table 1 and Additional file 1: Table S1). This pattern fits either with an ancient origin of DUF71 in Bacteria followed by numerous losses or, conversely, with a more recent acquisition followed by horizontal gene transfer (HGT) among bacterial lineages. To further investigate the evolutionary history of DUF71, we made a phylogenetic analysis of the homologs identified in the three Domains of Life. The resulting tree showed two divergent groups of sequences. The first group contains the eukaryotic and nearly all archaeal sequences (including the predicted yeast DPH6 (YLR143w) and *P*. *furiosus* PF0828), whereas the second encompasses all the bacterial sequences as well as the second copy found in a few archaeal genomes (Figure 4 and Additional file 3: Figure S3).

**Table 1:** **Taxonomic distribution of DUF71 homologs in archaeal and bacterial genomes**

**Phylum**	**Nb (%) genomes**	**Phylum**	**Nb (%) genomes**	**Phylum**	**Nb (%) genomes**
***Archaea***					
Crenarchaeota	37/37 (100%)	Korarchaeota	0/1 (0%)	Thaumarchaeota	2/2 (100%)
Euryarchaeota	79/79 (100%)				
***Bacteria***					
Acidobacteria	3/7 (42.9%)	Dictyoglomi	0/2 (0%)	Proteobacteria_Epsilon	0/64 (0%)
Actinobacteria	1/206 (0.5%)	Elusimicrobia	0/2 (0%)	Proteobacteria_Gamma	27/406 (6.7%)
Aquificae	0/10 (0%)	Fibrobacteres	0/2 (0%)	PVC_Chlamydiae	1/73 (1.4%)
Bacteroidetes	20/73 (27.4%)	Firmicutes	20/484 (4.1%)	PVC_Planctomycetes	3/6 (50%)
Caldiserica	0/1 (0%)	Fusobacteria	0/5 (0%)	PVC_Verrucomicrobia	0/4 (0%)
Chlorobi	0/11 (0%)	Gemmatimonadetes	0/1 (0%)	Spirochaetes	1/45 (2.2%)
Chloroflexi	5/16 (31.3%)	Ignavibacteria	0/1 (0%)	Synergistetes	0/4 (0%)
Chrysiogenetes	0/1 (0%)	Nitrospirae	1/3 (33.3%)	Thermodesulfobacteria	0/2 (0%)
Cyanobacteria	0/45 (0%)	Proteobacteria_Alpha	2/204 (1%)	Thermotogae	5/14 (35.7%)
Deferribacteres	0/4 (0%)	Proteobacteria_Beta	8/119 (6.7%)		
Deinococcus-Thermus	2/17 (11.8%)	Proteobacteria_Delta	1/48 (2.1%)		

The number of genomes per phylum containing at least one homolog of DUF71 is indicated.

This second group emerged from within the archaeal sequences of the first cluster and showed various contradictions with the currently recognized taxonomy because bacterial sequences from distantly related lineages appeared intermixed in the tree (Figure 4). These observations together with the extremely patchy distribution of DUF71 in bacteria strongly supports the hypothesis that the bacterial DUF71 was of archaeal origin and spread through this domain mainly by HGT. Interestingly, the second homologs present in a few archaeal genomes emerged from bacterial sequences, suggesting that secondary HGT occurred from Bacteria to Archaea allowing them acquiring a second DUF71 homolog.

In contrast, a phylogenetic analysis focused on archaeal and eukaryotic sequences strongly supported the separation between these two Domains (posterior probabilities (PP) = 1). Moreover it recovered the monophyly of most eukaryotic and archaeal major lineages (most PP > 0.95, Additional file 3: Figure S3), suggesting that DUF71 was present in their ancestors. However, as expected given the small number of amino acid positions analyzed (182 positions), the relationships among these lineages were mainly unresolved (most PP < 0.95) precluding the in-depth analysis of the ancient evolutionary history of DUF71 in Archaea and Eucarya (Additional file 3: Figure S3). Nevertheless, the wide distribution of DUF71 in these two Domains (even in highly derived parasites such as *Microsporidia*, *Cryptosporidium*, *Entamoeba* or *Nanoarchaeum equitans*, not shown) and its ancestral presence in most of their orders/phyla suggested that this gene was present in the last common ancestor of these two Domains. This inference does not imply, however, that no HGT occurred in these Domains. Indeed, some incongruence between the DUF71 phylogeny and the reference phylogeny of organisms [39] suggested putative cases of HGT. For instance, it was observed for the *Thermofilum pendens* DUF71 that robustly groups with Methanomicrobia (Euryarchaeota) and not with other Thermoproteales (Additional file 3: Figure S3).

**Figure 5:**
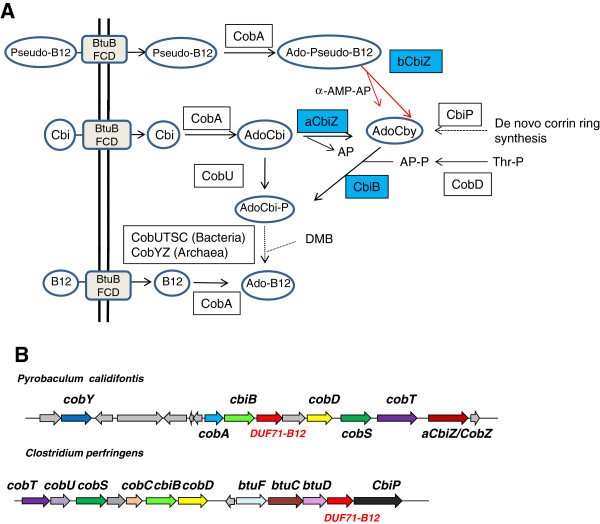
**Links between the DUF71 family and B12 salvage. **(**A**) Summary of cobinamide derivative salvage in Bacteria and Archaea; arrows with dotted lines denote multiple steps. (**B**) Typical examples of physical clustering of DUF71-B12 genes with B12 salvage genes in Archaea and Bacteria. Abbreviations: Pseudo-B12, adenosylpseudocobalamin; Cbi, Cobinamide; AdoCbi, adenosylCbi; AdoCbi-P, AdenosylCbi-phosphate; AdoCby, adenosylcobyric acid; AP; (R)-1-amino-2-propanol; AP-P, AP-phosphate; Thr-P, L-threonine-phosphate; DMB, 5,6-dimethylbenzimidazole; α-AMP-AP, α-adenylate-AP; CobU, ATP:AdoCbi kinase, GTP:AdoCbi-GDP guanylyltransferase; CobY, NTP:AdoCbi-P nucleotidyltransferase; CobA, ATP:co(I)rrinoid adenosyltransferase; aCbiZ, adenosylcobinamide amidohydrolase; bCbiZ, pseudo-B12 amidohydrolase; CbiB, cobyric acid synthetase; CobD, L-threonine phosphate decarboxylase; CobS, cobalamin (5-P) synthase; CobT, 5,6-dimethylbenzimidazole phosphoribosyltransferase; CobC or CobZ, alpha-ribazole-5′-phosphate phosphatase; cobY, adenosylcobinamide-phosphate guanylyltransferase; CbiP, cobyric acid synthase; BtuFCD, cobamide transporter subunits.

Because diphthamide is a modification specific to the archaeal and eukaryotic EF-2 proteins and bacteria lack all known diphthamide biosynthesis genes, we propose that cluster 1 in our phylogeny corresponds to *bona fide* Dph6 enzymes involved in diphthamide synthesis (Figure 4). This function therefore very likely represents the ancestral function of the whole DUF71 family. In contrast, bacteria do not synthesize diphthamide, suggesting that the bacterial DUF71 homologs and the few additional archaeal copies (cluster 2, Figure 4) are involved in another function, and thus a functional shift occurred after the HGT of an archaeal bona fide Dph6 to bacteria. Notably, these genes (including PF0295, the second DUF71 copy found in *P*. *furiosus*) are strongly clustered on the chromosome with vitamin B12 salvage genes. More precisely 75/102 are adjacent to vitamin B12 transporter genes (such as the BtuCDF genes) [40] and 18/102 are adjacent to *cbiB* genes encoding adenosylcobinamide-phosphate synthetase, an enzyme shared by the *de novo* and salvage pathways [41] (Figure 5A). This clustering data can be visualized in the “Duf71-B12” subsystem in the SEED database, and two typical clusters are shown in Figure 5B. On this basis, we hypothesize that the archaeal and bacterial DUF71 genes that cluster with B12 vitamin genes have a role in B12 metabolism.

Finally, some bacterial DUF71 proteins might also have other functions because a set of bacteria such as Clostridium perfringens have two or more DUF71 homologs (Figure 4 and Additional file 1: Table S1). The most extreme example is Dehalococcoides sp. CBDB1, which encodes five DUF71 homologs in its genome. In the case of C. perfringens ATCC 13124 and SM101, one homolog (YP_695745 and YP_698440) clusters both physically and phylogenetically (Figure 4 and 5A) with the B12 subgroup proteins, whereas the second homolog (YP_695178 and YP_698039) is related to Acinetobacter baumanii (Cluster 3, Figure 4) and is not found associated to gene clusters related to B12 salvage (data not shown).

Therefore, based on phylogenetic and physical clustering the DUF71 proteins were split into: the Dph6 and the DUF71-B12 subgroups that were annotated as such and captured in the “Diphthamide biosynthesis” and “Duf71-B12” subsystems in the SEED database.

### Predicting the function of members of the DUF71-B12 subgroup

As members of the DUF71-B12 subgroup clustered strongly with B12 transport genes and with *cbiB* (Figure 5B), we focused on the early steps on B12 salvage, which are quite diverse because several forms of cobamides [cobalamin-like or Cbl-like compounds] can be salvaged (Figure 5A). Cobinamide (Cbi) is adenylated after transport to form adenosylcobinamide (AdoCbi). In most bacteria, AdoCbi is directly phosphorylated by CobU before being transformed after several steps into adenosylcobalamin (AdoCbl or coenzyme B12), in which the lower ligand is 5,6-dimethylbenzimidazole (DMB) (see [42] for review) (Figure 5A). Archaea use a different salvage route in which AdoCbi is converted to adenosylcobyric acid (AdoCby), an intermediate of the *de novo* pathway, by an amidohydrolase, aCbiZ [43] (Figure 5A). AdoCby is then converted directly to adenosylcobinamide-phosphate (AdoCbi-P) by CbiB. Finally some bacteria have CbiZ homologs (bCbiZ) that hydrolyze adenosylpseudocobalamin (Ado-Pseudo-B12) [44], which contains an adenine instead of DMB as its lower ligand (Figure 1C and 5A).

**Figure 6:**
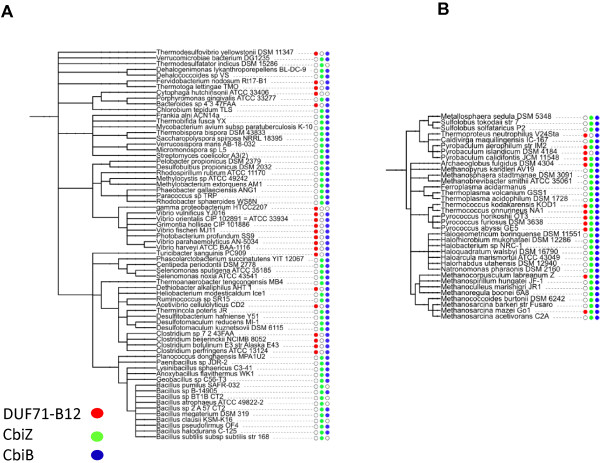
**Distribution of DUF71-B12, CbiZ and CbiB in bacterial (A) and archaeal genomes (B). **The trees are species tree constructed in iTol (itol.embl.de/), the presence and absence of the specific genes was derived from the “DUF71-B12” subsystem in the SEED database.

In order to gain insight into the possible function of DUF71-B12 family members, we analyzed the co-distribution pattern of CbiZ, CbiB and DUF71-B12 proteins in Archaea and Bacteria. Interestingly, to a few exceptions, all prokaryotic genomes encoding CbiB harbor either CbiZ or DUF71-B12 (Figure 6). However, in bacteria, there was strict anti-correlation between the DUF71-B12 and the CbiZ families (Figure 6A). This was not the case in Archaea where quite a few organisms (such as *P*. *furiosis* or *Methanosarcina mazei* Go1) harbored both families (Figure 6B). This distribution profile suggests that members of the DUF71-B12 subfamily fulfil the same roles as the bacterial CbiZ enzymes (bCbiZ), either by catalysing the same reaction (cleaving Ado-pseudo-B12 into AdoCby) or by providing another route to salvaging Pseudo-B12. This hypothesis would explain why bacteria would have one or the other while Archaea could carry both (Figure 6B), because archaeal CbiZ proteins have been predicted to lack pseudo-B12 cleavage activity [44].

Detailed analysis of the signature motifs of the two subfamilies reveal that the strictly conserved EGGE/DXE_188 _motif (*P*. *furiosus* PF0828 numbering) in Dph6 proteins is replaced by a ENGEF/YH_188 _ motif in the DUF71-B12 proteins (Additional file 3: Figure S2 and Additional file 3: Figure S4). In the Dph6 family, E188 is located near the predicted diphthine binding site and is predicted to be involved in catalysis (Figure 3B). The replacement of the strictly conserved E188 residue by a Histidine residue strongly suggest a change in the reaction catalyzed by the DUF71-B12 subfamily compared to the Dph6 family. The structure based comparison between the two subfamilies also strongly supports the hypothesis that their substrates are different, because all residues predicted to be involved in EF-2 binding (Figure 3B see section above) are different in the DUF71-B12 subfamily but mostly conserved within this subfamily (Additional file 3: Figure S2 and residues in green in Figure 3C). Residues that are conserved between the two DUF71 subfamilies (Additional file 3: Figure S2 and residues in blue in Figure 3C) are found around the phosphate groups of ATP, including S_12_, G_13_, G_14_, K_15_, D_16_, H_48_, and T_189 _(PF0828 sequence numbering) or belong to the C-terminal conserved sequence motif (EGGE/D-X-E188) such as G_182_, G_184_, G_185_, E_186_, F_187_ (Additional file 3: Figure S2 and Figure 3C). Further experimental studies will be required to determine whether DUF71-B12 proteins are Ado-pseudo-B12 amidohydrolases or have another role in Ado-pseudo-B12 salvage.

## Conclusions

Our detailed analyses of the DUF71 family members presented here provide an example of the power of comparative genomic approaches for solving important “missing genes” or “missing function” cases. These analyses simultaneously illustrate the difficulties inherent in accurately annotating gene families. On one hand, the evidence identifying a candidate for the missing Dph6 gene family derived from genomic evidence (mainly phylogenetic distribution and gene fusions) and post-genomic evidence (structure, co-expression analysis and genome-wide phenotype experiments) is so strong that it could be used as an example where the functional annotation of a protein of unknown function could be derived from comparative genomic alone. On the other hand our analyses show that a subgroup of the DUF71 family is most certainly involved in a metabolic pathway unrelated to diphthamide synthesis and that transferring functional annotations from homology scores alone would be inappropriate in this case. We believe that this integrated functional annotation approach will play an important role in future pipelines for annotation of protein families.

## Competing interests

The author(s) declare that they have no competing interests.

## Authors’ contributions

VdC-L conducted the comparative genomic analysis and made the functional predictions. CB-A performed the phylogenetic analysis. FF, LT and JFH did the structural analysis. All authors participated in writing/reviewing the manuscript. All authors read and approved the final manuscript.

## Reviewers’ comments

Reviewer number 1: Arcady Mushegian

**Stowers Institute for Medical Research, 1000 E 50**^**th**^**Street, Kansas City, Missouri 64110**

The study by de Crecy-Lagard and co-authors pinpoints the DUF71/COG2102 asthe most likely archaeal/eukaryotic ATP-dependent diphthine-ammonia ligase,the so far unaccounted-for enzyme in the pathway of diphtamide biosynthesis, which pathway is responsible for the formation of unique derivative of the conserved histidine within the translation elongation factor 2. A distinct subfamily of this protein family appears to play another role in bacteria and a subset of archaea, most likely in the salvage of an intermediate of cobalamine biosynthesis. The evidence presented in the paper consists of genome context information, sequence-structure prediction and the data from yeast concerning gene expression and chemical-genomics profiling. Taken together, the evidence seems compelling to me. The data from yeast represent partial functional validation of predictions made for prokaryotes. I would recommend only to tone down the suggestion that all this is a “novel paradigm” in analysis of gene function: researchers have been inferring gene functions from phenotypes, as well as from directly detected changes in genotype, for a long, long time, and the current study is a logical extension of these approaches. What is different in the last 15 years is that we can compare these properties across many species with completely sequenced genomes; but even this is a logical extension of the previous work (compare, for example, with work from Yanofskyand Jensen labs on biosynthesis of aromatic amino acids) - it was not any prescription of a previous scientific paradigm that constrained the work, but rather the lack of the data.

Response: *The references to a “novel paragdim” were eliminated in the abstract and the introduction as suggested.*

Reviewer number 2: Michael Galperin

NCBI, NLM, NIH Computational Biology Branch, 8600 Rockville Pike MSC 6075, Building 38A, Room 6N601, Bethesda, MD 20894-6075

The paper by de Crecy-Lagard and colleagues is a fine example of using comparative genomics to patch the remaining holes in the metabolic pathways. The key conclusion of this work, prediction of the participation of the members of the DUF71/COG2102 family in diphtamide biosynthesis in archaea and eukaryotes and in B12 metabolism in some bacteria and archaea, is extremely convincing and hardly even needs an experimental verification. The second conclusion, that ammonia used in the diphthine ammonia lyase-catalyzed reaction in different organisms could use generated by two different enzymes, asparagine synthetase and the RidA domain, also sounds convincing. However, proving beyond reasonable doubt that DUF71/COG2102 family members with their ATP-pyrophosphatase activity comprise the key part of diphthine ammonia lyase does not prove that they are the only subunits of this enzyme. Even if the proposed reaction scheme (Figure 1B) is correct, there still might be a need for a ligase subunit that couple removal of the AMP moiety from EF2 with its amidation. There is a definite possibility that DUF71/COG2102 family members catalyze all these individual reactions, e.g. using its unique C-terminal 100-aa domain, but that would have to be proven experimentally. The reported involvement of the likely scaffold protein YBR246w (DPH7) appears to support the idea that diphthine ammonia lyase consists of more than one type of subunits. Otherwise, it is a great paper that vividly demonstrates the power of comparative-genomics approaches.

We added a phrase stating that “even if one cannot rule out at this stage that other catalytic subunits yet to be identified may also be required”.

Reviewer number 3: L. Aravind

NCBI, NLM, NIH Computational Biology Branch, 8600 Rockville Pike MSC 6075, Building 38A, Room 6N601, Bethesda, MD 20894-6075

This work uses contextual information to identify the diphthine-ammonia ligase in archaea and eukaryotes. It also shows that the yeast protein YBR246W is indeed not the correct ligase, but rather the MJ0570-like PP-loop ATPases. The authors also show that this family has been transferred to certain bacteria where they infer that it is likely to have undergone a functional shift to participate in B12 salvage. They cautiously propose that it might function as a replacement for CbiZ to function as an amidohydrolase (the reverse of the typical PP-loop ATPase reaction) as against a ligase. The conclusions are definitive and the article makes a useful contribution to the understanding of protein modification and cofactor biosynthesis. This said, there are certain issues with the current form of the article that authors necessarily need to address in their revision: 1) (pg 8) The authors state that the MJ0570-like enzymes have a HUP domain followed by a distinct C-terminal domain. They do not explain the meaning of this properly nor cite the reference of the paper (PMID: 12012333) pertaining to the HUP domains where this family was identified as a PP-loop ATPase, along with the observations (Table 1 in that reference) that it has a primarilyarchaeo-eukaryotic phyletic pattern, and that eukaryotic versions might be fused to two C-terminal domains of the YabJ-like chorismate lyase fold (now termed RidA). It should be stated that the N-terminus is a PP-loop ATPase domain of the HUP class of Rossmannoid domains - not all HUP domains are ligases - only the PP-loop and the HIGH nucleotidyltransferases . This clarifies that it is related to other ATP-utilizing amidoligases such as NAD synthethase, GMP synthetase and asparagine synthetase. This would place their inferred amidoligase activity in the context of comparable, known amidoligase activities of related enzymes. In fact it would be advisable to place the fact that these are PP-loop enzymes in the abstract itself.

The following sentence was added: “This family had previously been previously described as a PP-loop ATPase of unknown function containing a Rossmannoid class HUP domain (Aravind et al. 2002).” A reference to the PP-loop ATPase family was added in the abstract as requested. A reference to the same work was added when talking about the RidA fusion. For the phylogenetic distribution the results presented here are a bit different from the previous study because many more genome are available after 10 years and we show that the family is also bacterial.

2) The authors persistently refer to the domain as DUF71. This name is no longer current in Pfam and it has long been recognized as mentioned in the reference noted above that these proteins are not “domains of unknown function” but PP-loop ATPases. The domain is correctly termed ATP_bind_4 (PF01902) in Pfam. This Pfam (not the misleading DUF71) name and Pfam number should be indicated with just a statement in the introduction that it was formerly DUF71.

This domain is currently called “Domain of unknown function DUF71, ATP-binding domain” in the InterPro database (IPR002761) even if it is called ATP_bind_4 (PF01902) in Pfam. It is much shorter to use (as well as easier for the reader to follow) the DUF71 abbreviation rather than the ATP_bind_4 abbreviation. We therefore prefer to keep DUF71. We however introduced a statement giving the different names of this domain in the InterPro, Pfam and COG databases at the end of the introduction.

*3*) *The authors apparently have a misapprehension regarding the Methanohalophilus mahii protein both in the text and the domain architecture rendered in the figure*. *First*, *these proteins have two N-terminal domains fused tothe MJ0570-like module: namely aN-terminal class-II glutamineamidotransferase (GAT-II, e.g. see PMID: 20023723) and second PP-loop ATPase domain thereafter (i.e. one related to asparagine synthetase). This GAT domain as in the case of other PP-loop enzymes could supply ammonia by cleaving it off glutamine. But this does not explain which PP-loop domain utilizes it. In the case of the Asn-synthetase it is used by the cognate PP-loop domain. In this case the presence of two PP-loop domains suggests that it is either utilized by both for different reactions or else the second domain does not receive the NH3 from this GAT. This also leads to the question what reaction is the Asn synthetase like PP-loop domain catalyzing*? 

Quality of written English: Acceptable

The source of the confusion came from the fact that the Asn Synthase domain (AsnB) contains two domains the GAT-II domain and the Asn_Synthase_B_C PP-Loop ATPase domain. Both the figure and the text were modified to avoid the confusion. Based on the reviewer’s comments the sentence discussing the potential role of the AsnB domain was modified as follows: “This domain organization strongly suggests that in this subset of enzymes, the hydrolysis of glutamine catalyzed by the fused GAT-II domain could provide the NH_3 _moiety to both the DUF71 and the Asn_Synthase_B_C enzymes.”

4) Based on phyletic complementarity the authors suggest that bacterial CbiZ might be displaced by the bacterial MJ0570-like enzymes. This seems unusual - Why utilize a PP-loop ATPase for the reverse reaction, i.e. amidohydrolase? Typically there is little overlap between the families involved in amidohydrolase as opposed to ATP-dependent ligase activity. Of the almost 12 distinct major inventions of amidoligase activity, hardly any representatives of these superfamilies have been reused as amidohydrolases. So do the authors note anything special in the case of the bacterial representatives that might support such a functional shift?

This hypothesis is derived from phylogenetic distribution and it is not unprecedented that ligases and hydrolases are found in the same family (see example in PMID:12359880). However, we agree that this hypothesis derives mainly from phylogenetic patterns analysisand beyond the differences in the predicted substrate binding pocket found in the DUF71-B12 family we did not identify specify changes that could point to a shift to hydrolase, hence our caution in our prediction as stated in the text.

Quality of written English: Acceptable

## Supplementary Material

Additional file 1 **Table S1. **Genbank RefSeq identities and corresponding organisms for all proteins used in the phylogenies. Click here for file

Additional file 2 ** Table S2. **GO Term Enrichment Spell analysis (http://imperio.princeton.edu:3000/yeast) with YLR143w as input. Click here for file

Additional file 3 **Figure S1. **Top 10 interactors with YLR143W by homozygous co-sensitivity in S. cerevisiae (from the Yeast fitness database http://fitdb.stanford.edu/fitdb.cgi?query=YLR143W). **Figure S2** Multiple sequence alignment of selected Dph6 family and DUF71-B12 family sequences generated using the Multialin platform (http://multalin.toulouse.inra.fr/multalin/) Strictly conserved residues between the two families are in red. Residues conserved only in the Dph6 family are boxed in green. Residues found around the phosphate group of ATP are noted by red arrows. Secondary structural elements, yellow rectangles for α-helix and cyan arrows for β-strand, shown above the alignment, are from the crystal structure of P. furiosus_Dph6 (PF0828) (PDB id: 3RK1). **Figure S3** Bayesian tree of archaeal and eukaryotic Dph6 sequences. The scale bar represents the average number of substitutions per site. Number at nodes represent posterior probabilities. For clarity only values greater than 0.85 are indicated. **Figure S4** (Top) Sequence logo derived from 95 Dph6 sequences extracted from Diphthamide subsystem in SEED. The E188 reside (PF0828 numbering) is located at position 10 in the logo. (Bottom) Sequence logo derived of the corresponding region derived from 102 DUF71-B12 sequences extracted from the DUF71-B12 subsystem in SEED. Both logos were made at http://weblogo.berkeley.edu/logo.cgi based on clustalw derived alignments. Click here for file
