# Supplementary material for: Comparative genomic analysis of the DUF71/COG2102 family predicts roles in diphthamide biosynthesis and B12 salvage
Source: Biol Direct. 2012 Sep 26;7:32. doi: 10.1186/1745-6150-7-32 (PMC3541065; doi:10.1186/1745-6150-7-32)
Supplement: Additional file 3 — Figure S1. Top 10 interactors with YLR143W by homozygous co-sensitivity in S. cerevisiae (from the Yeast fitness database http://fitdb.stanford.edu/fitdb.cgi?query=YLR143W). Figure S2 Multiple sequence alignment of selected Dph6 family and DUF71-B12 family sequences generated using the Multialin platform (http://multalin.toulouse.inra.fr/multalin/) Strictly conserved residues between the two families are in red. Residues conserved only in the Dph6 family are boxed in green. Residues found around the phosphate group of ATP are noted by red arrows. Secondary structural elements, yellow rectangles for α-helix and cyan arrows for β-strand, shown above the alignment, are from the crystal structure of P. furiosus_Dph6 (PF0828) (PDB id: 3RK1). Figure S3 Bayesian tree of archaeal and eukaryotic Dph6 sequences. The scale bar represents the average number of substitutions per site. Number at nodes represent posterior probabilities. For clarity only values greater than 0.85 are indicated. Figure S4 (Top) Sequence logo derived from 95 Dph6 sequences extracted from Diphthamide subsystem in SEED. The E188 reside (PF0828 numbering) is located at position 10 in the logo. (Bottom) Sequence logo derived of the corresponding region derived from 102 DUF71-B12 sequences extracted from the DUF71-B12 subsystem in SEED. Both logos were made at http://weblogo.berkeley.edu/logo.cgi based on clustalw derived alignments. [file 1745-6150-7-32-S3.pdf]

Supplemental Figure 1

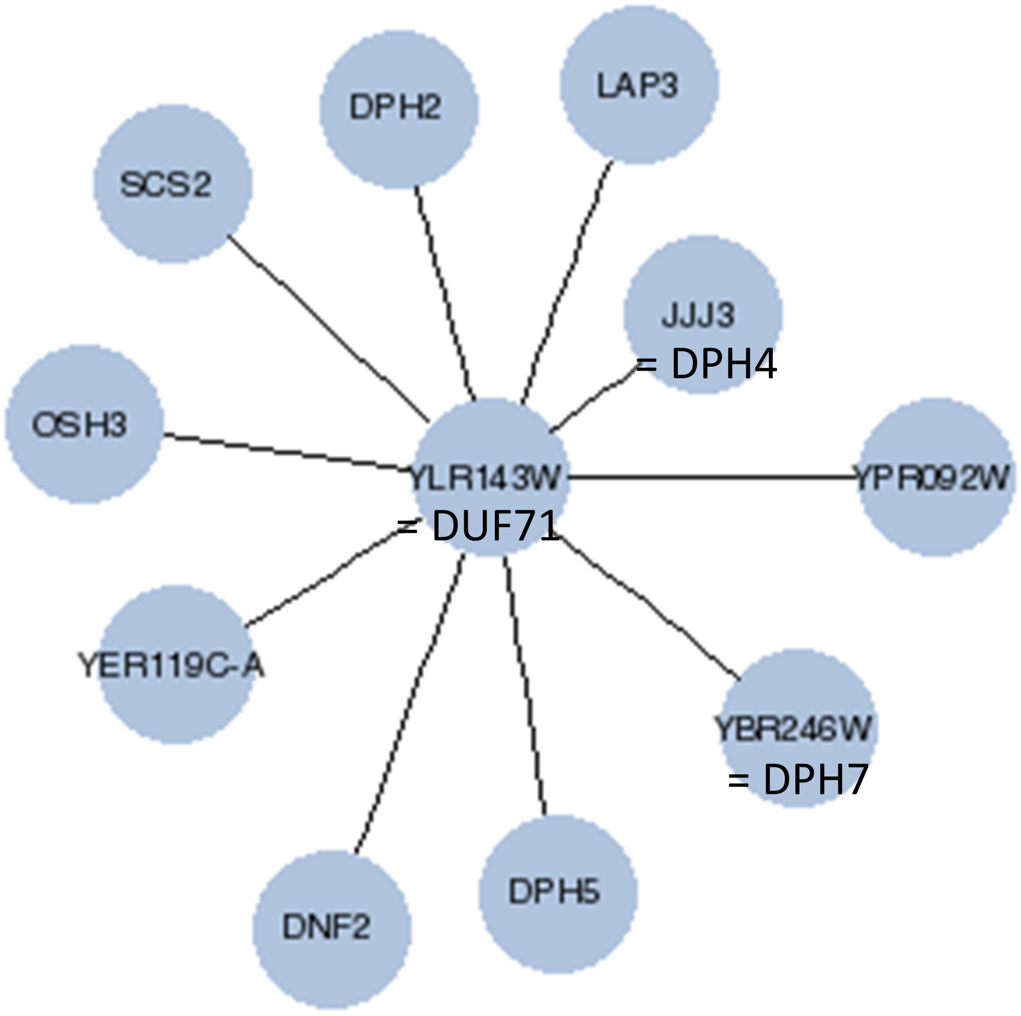

Top 10 interactors with YLR143W by homozygous co-sensitivity in S. cerevisiae (from the Yeast fitness database http://fitdb.stanford.edu/fitdb.cgi?query=YLR143W).

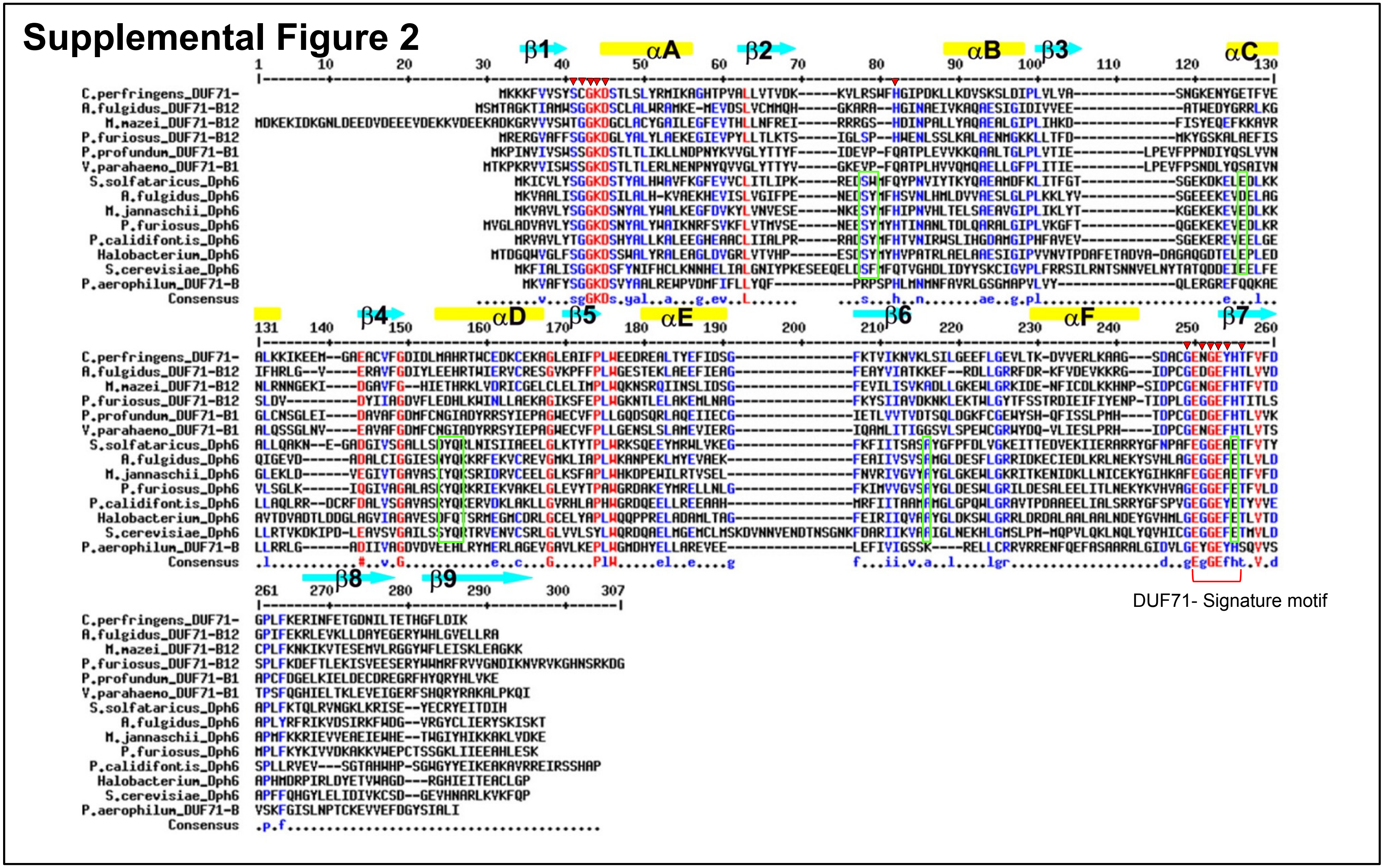

Multiple sequence alignment of selected Dph6 family and DUF71-B12 family sequences generated using the Multialin platform (http://multalin.toulouse.inra.fr/multalin/) Strictly conserved residues between the two families are in red. Residues conserved only in the Dph6 family are boxed in green. Residues found around the phosphate group of ATP are noted by red arrows. Secondary structural elements, yellow rectangles for α-helix and cyan arrows for β-strand, shown above the alignment, are from the crystal structure of P. furiosus_Dph6 (PF0828) (PDB id: 3RK1).

Supplemental Figure 3

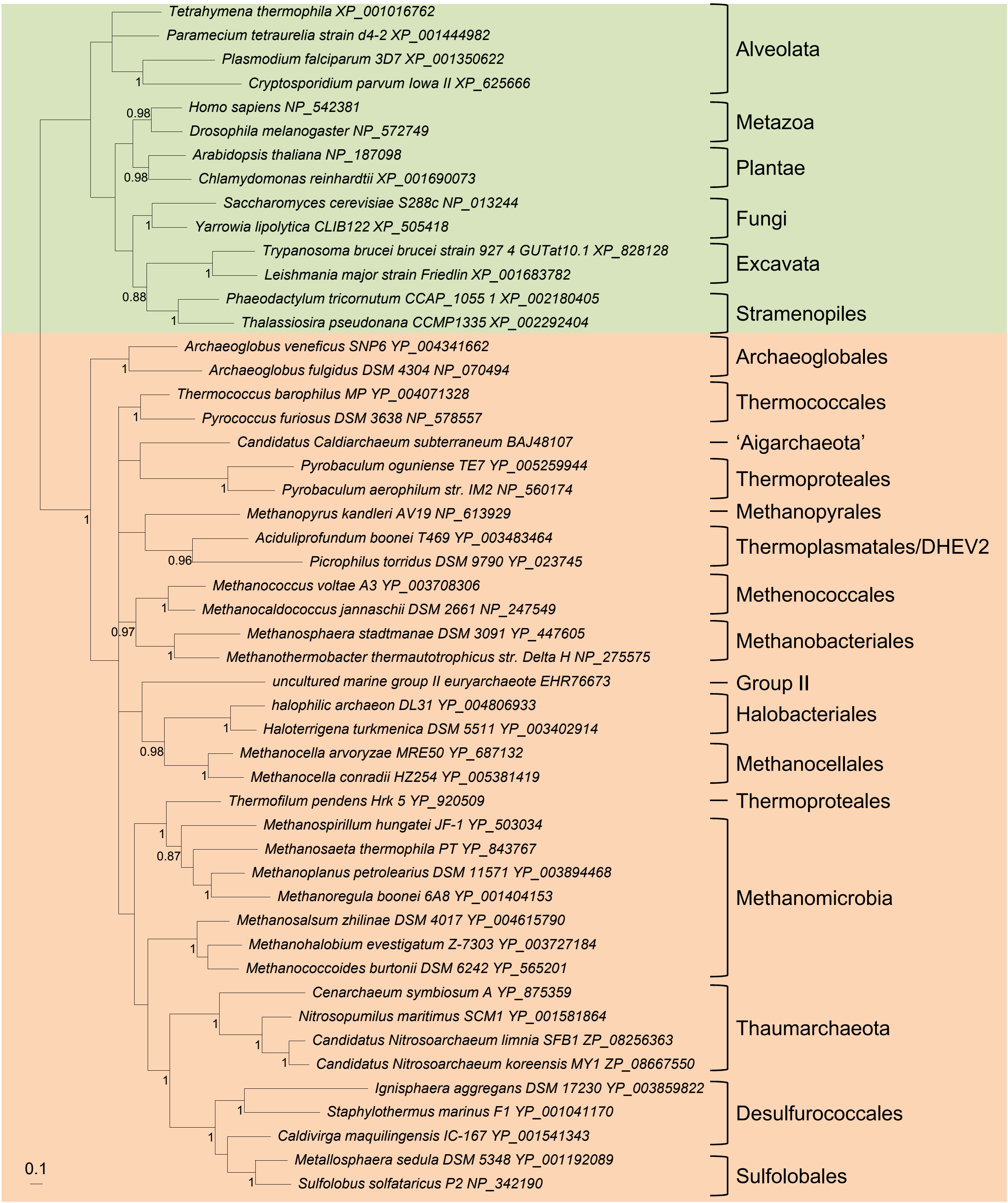

Bayesian tree of archaeal and eukaryotic Dph6 sequences. The scale bar represents the average number of substitutions per site. Number at nodes represent posterior probabilities. For clarity only values greater than 0.85 are indicated.

Supplemental figure 4

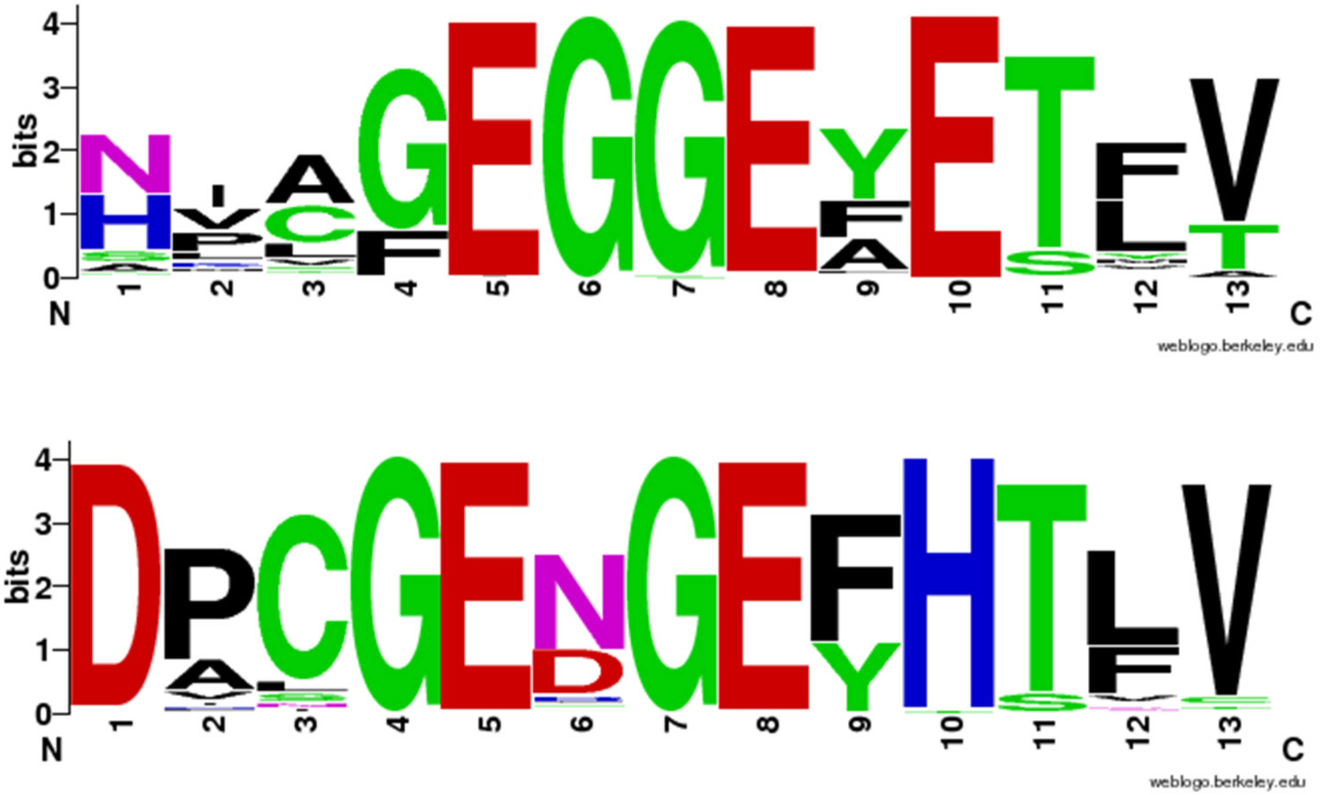

(Top) Sequence logo derived from 95 Dph6 sequences extracted from Diphthamide subsystem in SEED. The E188 reside (PF0828 numbering) is located at position 10 in the logo. (Bottom) Sequence logo derived of the corresponding region derived from 102 DUF71-B12 sequences extracted from the DUF71-B12 subsystem in SEED. Both logos were made at http://weblogo.berkeley.edu/logo.cgi based on clustalw derived alignments.
